# Trends of interventional radiology procedures during the COVID-19 pandemic: the first 27 weeks in the eye of the storm

**DOI:** 10.1186/s13244-020-00938-8

**Published:** 2020-12-09

**Authors:** Guo Yuan How, Uei Pua

**Affiliations:** Tan Tock Seng Hospital, 11 Jalan Tan Tock Seng, Singapore, 308433 Singapore

**Keywords:** Radiology, Interventional, COVID-19, Singapore

## Abstract

**Objectives:**

While the Novel Coronavirus (COVID-19) pandemic looks to persist, institutions promote delaying procedures. Understanding trends and demands of interventional radiology (IR) procedures in the infected and COVID-free populations are needed in long-term planning. We detail IR procedure trends in the first 27 weeks of the pandemic and compare with the pre-pandemic era.

**Methods:**

In this IRB approved retrospective electronic case review, all IR patients in our institution from 1 January to 9 July 2020, the same period in 2019 pre-pandemic and the Severe Acute Respiratory Syndrome (SARS-CoV) outbreak were included. IR procedures were classified using Interventional Radiology—Procedure Acuity Scale (IR-PAS) and category of IR procedures. Along with descriptive frequencies, the Mann–Whitney *U* test and Chi-square test of independence were performed.

**Results:**

During the pandemic, 3655 IR procedures were performed compared to 3851 procedures pre-pandemic. No statistically significant difference in weekly IR caseloads across IR-PAS tiers between both periods (*p* = .088) and category of procedure (*p* = .054) were noted. General intervention procedures remained the largest proportion and musculoskeletal procedures the minority, in both periods. More general intervention radiology and oncology procedures were performed during the COVID-19 pandemic compared to the SARS-CoV outbreak. Thirty-four (0.93%) IR procedures were performed on 30 COVID-19 patients. There was no IR procedure-related COVID-19 cross-transmission.

**Conclusions:**

Demand for IR procedures among COVID-free patients remains high, and IR procedures involving COVID-19 represents a fraction of the IR caseload. A sustainable model in providing timely IR services to COVID-free patients needs to be considered.

## Key points

- The demand for IR procedures for COVID-free patients remains high during this pandemic.
- The optimization of healthcare resources and adherence to standard infection control protocols by an IR department can yield similar operational capacity compared to pre-pandemic periods.
- Given the potential prolongation of the COVID-19 pandemic and future infectious diseases outbreaks, more sustainable IR procedural guidelines should be considered rather than active postponement of treatments.

## Introduction

Singapore was one of the first countries outside of China to be affected by the Novel Coronavirus (COVID-19) [1], with the first confirmed case detected on the 23rd January 2020, and a surge in cases 3 months later [2]. At the time of writing, a total of 47,126 confirmed COVID-19 cases, with a single-day peak of 1400 new cases in mid-April 2020 [3]. COVID-related mortality, however, remains one of the lowest in the world at 0.1% (27 COVID-related deaths) [4].

Like during the Severe Acute Respiratory Syndrome (SARS-CoV) outbreak in 2003 [5], our hospital was designated as the main acute care center for the COVID-19 pandemic. Our institution is a 1,700-bed tertiary academic hospital and is co-located with the National Centre for Infectious Disease (NCID), a purpose-built facility for infectious diseases, with an additional 330 beds, including 30 intensive care unit beds [6]. The interventional radiology (IR) service provides IR coverage to both the main hospital and the NCID, the latter housing a dedicated negative pressure angiography suite, CT fluoroscopy and ultrasound procedure rooms.

At the peak of the pandemic, our inpatient bed surge capacity was increased to 1475 beds for COVID-19 patients in NCID and the main hospital combined, with another estimated 700 beds available for COVID-free patients. This represented a 30% increase in operational beds compared to the pre-pandemic period. Our dedicated COVID-19 emergency department saw a single-day peak attendance of 520 patients in March 2020 for COVID-related attendance. Throughout the pandemic, our institution catered for up to 70% of all COVID-19 patients requiring hospitalization, with the highest daily inpatient admission of 900 patients in April 2020.

Literature detailing personal protection, workflow and resource management specific to COVID-19 exist [7–11]. However, literature describing the actual impact on IR services and COVID-free patient care remains limited [12, 13]. While deliberate postponement of "non-essential" procedures would be intuitive in the onset of any major outbreak, this approach is not sustainable in a protracted pandemic. The negative impact of pandemic-related treatment delays on COVID-free patients are increasingly being recognized [14–16], and poses many challenges for the post-pandemic recovery. Understanding the healthcare demands of COVID-free patients during this pandemic will aid planning for a prolonged pandemic and future outbreaks.

Throughout the pandemic, IR services needed to balance between infection risk and resource optimization, against the delay of care for COVID-free patients [14–16]. Guidelines for procedural prioritization and acceptable delays such as the Procedure Acuity Scale (IR-PAS), by The Society of Intervention Radiology [17], which was adapted from the American College of Surgeons Elective Surgery Acuity Scale, were developed [18]. The American College of Surgeons also published COVID-19 Guidelines for Triage of Vascular Surgery Patients, relevant to endovascular treatments [19]. These references aided our strategy in providing IR service on two separate fronts; maintaining IR services to COVID-free patients and IR support to COVID-19 patients. This study aims to review the trend of IR procedures in our institution during the first 27 weeks of the pandemic compared to the pre-pandemic era.

## Materials and methods

**Fig. 1:**
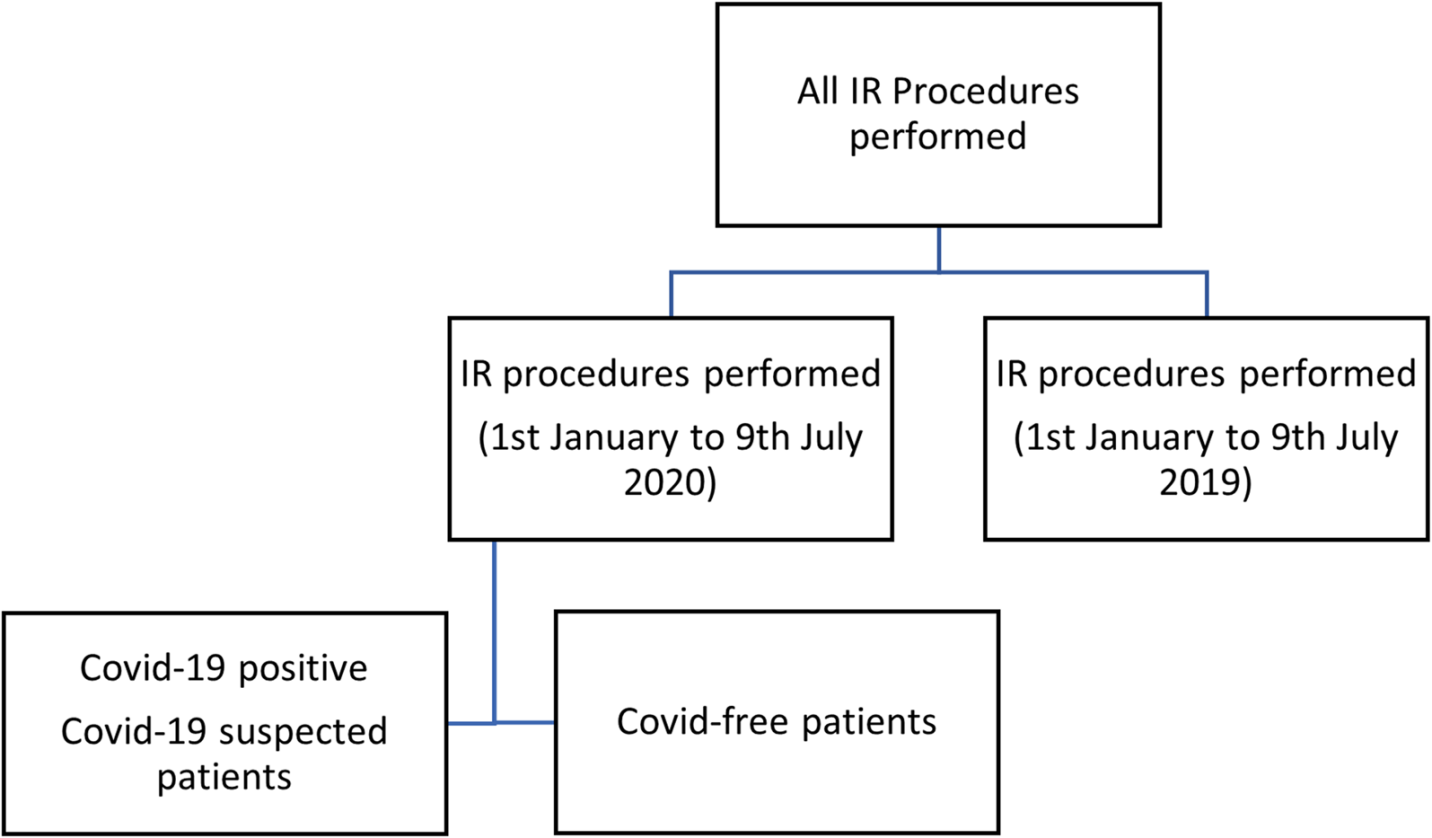
Flow diagram: flow diagram describing the recruitment of patients into the study. The study period was chosen to include the earliest official notice of COVID-19’s emergence by the World Health Organization up till the appropriate incubation period after the nation's eight-week circuit breaker. The corresponding pre-pandemic period in 2019 was chosen as it contained the traditional annual lull periods due to major cultural holidays in the country

This is an institutional ethics board-approved study. Waiver of consent was approved. We retrospectively reviewed the electronic medical records of all patients who underwent IR procedures in our institution from the 1st January 2020 to 9th July 2020 (COVID-19 pandemic) and the corresponding period in 2019 (pre-pandemic) (Fig. 1). The study period was specifically chosen to include the earliest official notice of COVID-19’s emergence by the World Health Organization [20] up till the appropriate incubation period after the nation's eight-week circuit breaker (heightened safe distancing measures, also commonly known as "lock-down") was lifted [21]. The corresponding pre-pandemic period was chosen as it contained the traditional annual lull periods due to major cultural holidays in the country. Specific filters were applied in on Picture Archiving and Communication system to identify patients who underwent IR procedures, and their online electronic medical records were reviewed. IR procedures on COVID-19 patients were identified using the NCID database. Cases of cross-transmission between patients and IR medical staff related to IR procedures were also obtained from our hospital surveillance system. No exclusion criteria were defined.

The trend of IR procedures was analyzed for any difference between the pre-pandemic and the COVID-19 pandemic period, using the IR-PAS classification (quantitative) into the three distinct tiers according to the procedure's acuity (Table 1), and broadly classified into four categories of IR procedures (qualitative). A table detailing all 3 tiers of the IR-PAS classification can be found in the Additional file 1. The four categories of IR procedures were: Vascular intervention, Interventional oncology, Musculoskeletal intervention, and General intervention (Table 2). Vascular procedures included treatment for peripheral vascular disease, dialysis access dysfunction, and aortic repair. Oncological procedures included diagnostic biopsies, tumor ablation, and transarterial therapies. Musculoskeletal procedures include spine augmentations and various joint injections. General interventional procedures consist of central line insertions (dialysis catheters, chemotherapy ports, etc.), emergency embolization, drainage procedures, nephrostomies, and biliary/gastrointestinal/genitourinary tract stenting. A comparison of the IR procedures performed on COVID-19 patients was made to our prior experience during the SARS-CoV outbreak [5]. All patients were screened and individuals fulfilling the relevant criteria were tested for COVID-19. COVID-19 cases were all confirmed using polymerase chain reaction in accordance with our institutional guidelines [22].

**Table 1:** (2020 and 2019: IR procedures classified based on IR-PAS classification)

		Frequencies				Mann–Whitney test		
		Median	SD	Variance	Range	Mann–Whitney *U*	Standardized test statistic	Asymptotic sig. (2-sided test)
Total	2020	133.00	22.175	491.739	93 (74–167)	288.000	− 1.705	0.088
	2019	142.50	27.075	733.073	137 (25–162)			
Tier 1	2020	27.00	6.299	39.676	23 (14–37)	419.000	0.443	0.658
	2019	26.00	6.142	37.729	31 (8–39)			
Tier 2	2020	57.00	12.556	157.656	49 (26–75)	301.000	− 1.492	0.136
	2019	64.50	13.492	182.025	64 (15–79)			
Tier 3	2020	48.00	8.871	78.698	35 (29–64)	293.000	− 1.624	0.104
	2019	53.00	10.423	108.639	45 (25–70)			

Weekly records of all IR procedures during the COVID-19 pandemic (period specified) and pre-pandemic (2019), classified based on the IR-PAS classification. Evaluation of differences in median weekly caseload across all IR-PAS tiers between the two time periods. No statistically significant differences are noted in the weekly median number of IR procedures performed across all IR-PAS tiers between the two time periods

*p < 0.05

**Table 2:** (2020 and 2019: categories of IR procedures)

			Year		Total	Chi-square test		Symmetric measures
			2020 (COVID-19 pandemic)	2019 (pre-pandemic)		Pearson Chi-Square	*df*	Cramer's *V*
Type of procedure	Vascular intervention (dialysis access angioplasties, lower limb angioplasties, aortic repair etc.)	Count	665	745	1410	7.648 (*p* = 0.054)	3	0.032 (*p* = 0.054)
		% within type of procedure	47.2%	52.8%	100.0%			
		% within year	18.2%	19.3%	18.8%			
		% of total	8.9%	9.9%	18.8%			
	Interventional oncology (intra-arterial therapy, tumor ablation, biopsy etc.)	Count	441	502	943			
		% within type of procedure	46.8%	53.2%	100.0%			
		% within year	12.1%	13.0%	12.6%			
		% of total	5.9%	6.7%	12.6%			
	Musculoskeletal intervention (spine augmentation, joint injections etc.)	Count	15	28	43			
		% within type of procedure	34.9%	65.1%	100.0%			
		% within year	0.4%	0.7%	0.6%			
		% of total	0.2%	0.4%	0.6%			
	General intervention radiology (central lines, Para/thoracentesis, percutaneous nephrostomy, GI/GU stenting etc.)	Count	2534	2576	5110			
		% within type of Procedure	49.6%	50.4%	100.0%			
		% within year	69.3%	66.9%	68.1%			
		% of total	33.8%	34.3%	68.1%			
Total		Count	3655	3851	7506			
		% within type of procedure	48.7%	51.3%	100.0%			
		% within year	100.0%	100.0%	100.0%			
		% of total	48.7%	51.3%	100.0%			

Weekly records of all IR procedures during the COVID-19 pandemic (period specified) and pre-pandemic (2019), classified based on the type of IR procedure. Evaluation of differences in median weekly caseload across all the four main categories of IR procedures between the two time periods. No statistically significant differences are noted in the weekly median number of IR procedures performed across all categories between the two time periods

A commercially available statistics program (IBM SPSS Statistics Grad Pack 27.0) was used for analysis. Statistical tests were two-sided. The Mann–Whitney U test was used to determine differences in the median number of IR procedures performed across all IR-PAS tiers during the COVID-19 pandemic and pre-pandemic period. The Chi-square test of independence was performed to examine the relation between the categories of IR procedures and the time periods (pre-pandemic and pandemic). A *p* value of < 0.05 was considered significant.

## Results

**Fig. 2:**
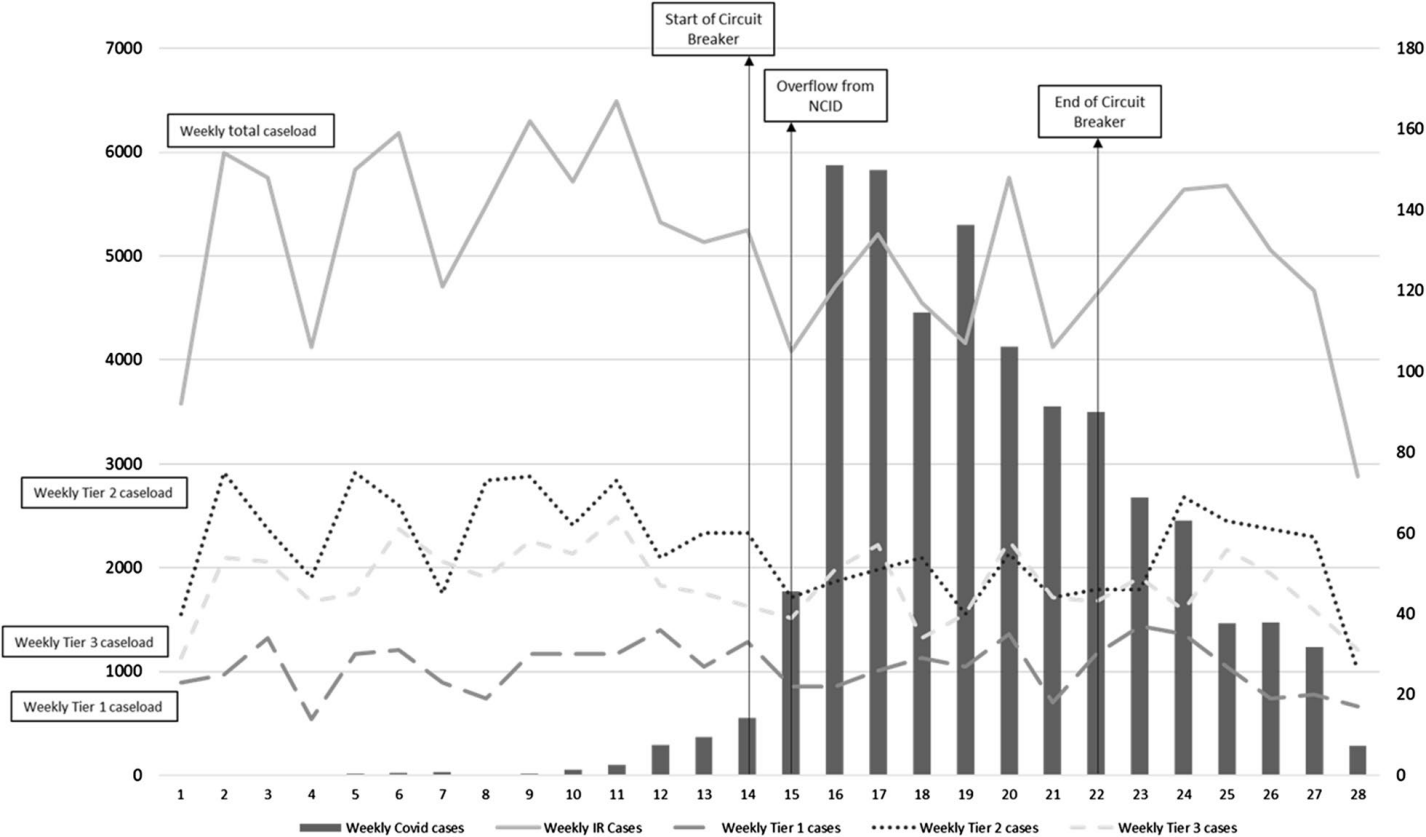
Weekly number of COVID-19 cases with weekly IR procedures performed classified based on IR-PAS classification: graphs describing the weekly number of COVID-19 patients in Singapore (with labels demarcating key events), and the weekly number of IR procedures performed across all IR-PAS tiers

**Fig. 3:**
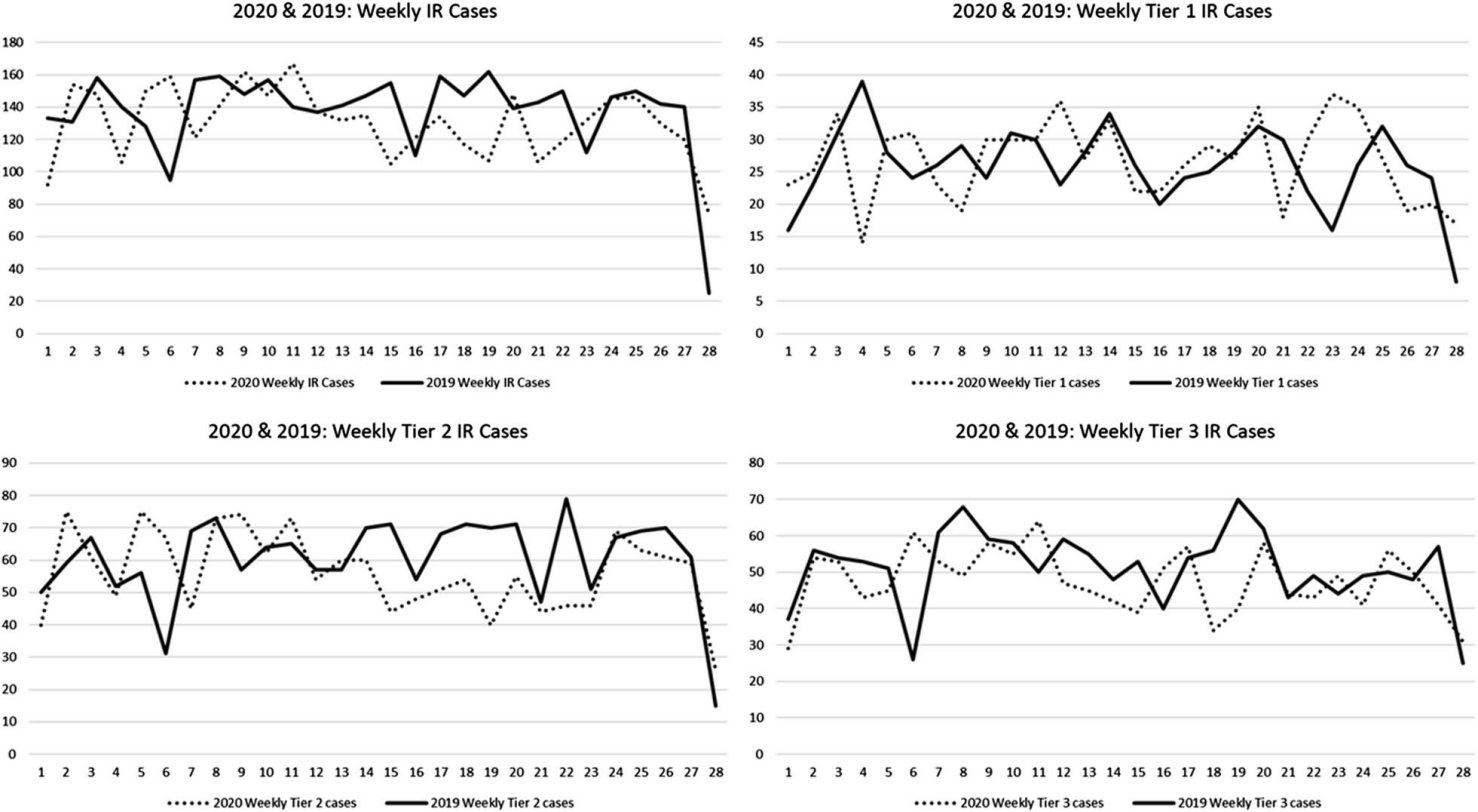
2020 and 2019: weekly number of IR procedures based on IR-PAS classification: Graphs illustrating the weekly number of IR procedures performed across all tiers, during the COVID-19 pandemic (2020) and pre-pandemic (2019)

There was a 5.1% decrease in the total IR procedures performed during the pandemic (*n* = 3655) versus the pre-pandemic period (*n* = 3851). Distributions of the weekly number of IR procedures were similar across all IR-PAS tiers, as assessed by visual inspection (Figs. 2, 3). There was no statistical significance between the median weekly IR procedures (pandemic 133.00, SD = 22.175 vs. pre-pandemic 142.50, SD = 27.075, *p* = 0.088). No statistical significance was found when comparing the weekly median number of IR procedures in the different Tiers; Tier 1: 27.00 (SD = 6.299) vs 26.00 (SD = 6.142), Tier 2:57.00 (SD = 12.556) vs 64.50 (SD = 13.492), Tier 3: 48.00 (SD = 8.871) vs 53.00 (SD = 10.423), during the COVID-19 pandemic and pre-pandemic period respectively. The median IR procedures performed were not statistically significant between both periods across all IR-PAS tiers (Table 1).

When comparing the categories of IR procedure between the pre-pandemic and the pandemic period, the trend was similar, with General IR (pandemic 69.5% vs. pre-pandemic 66.9%) procedures accounting for the majority of IR procedures, followed by vascular intervention IR (pandemic 18.2% vs. pre-pandemic 19.3%), interventional oncology IR (pandemic 12.1% vs. pre-pandemic 13.0%) and musculoskeletal intervention IR (pandemic 0.4% vs. pre-pandemic 0.7%). All expected cell frequencies were greater than 5. No statistically significant association between the time periods and categories of IR procedures performed were noted (*p* = 0.054). The association was small (Cohen, 1988), Cramer's V = 0.032. The results are summarized in Table 2.

COVID-19 patients constitute only 0.93% of all IR procedures, with 34 IR procedures performed on 30 patients. The most common procedures being peripherally inserted central catheters for vascular access (*n* = 7) and therapeutic para/thoracocentesis (*n* = 7) (Table 3), similar to that during the SARS-COV outbreak [5]. There was no record of COVID-19 cross-transmission between patients or to medical staff in all the IR procedures during the study period.

**Table 3:** IR procedures performed during the COVID-19 pandemic at NCID and the SARS-CoV outbreak

Type of procedure	Procedure	COVID-19 Pandemic (2020) *n* (%) *n* = 34	SARS-CoV Outbreak (2003) *n* (%) *n* = 28
Vascular intervention	Permanent catheter insertion/change	3 (8.8%)	4 (14.3%)
	Peripherally inserted central catheter	7 (20.6%)	8 (28.6%)
	Thoracic Endovascular Aortic Repair (TEVAR)	1 (2.9%)	
	Retrieval of vascular central catheter		1 (3.6%)
	Inferior vena cava filter insertion		1 (3.6%)
Interventional oncology	Axillary biopsy	1 (2.9%)	
	Hepatic tumor embolization	1 (2.9%)	
General intervention radiology	Para/thoracentesis	7 (20.6%)	12 (42.9%)
	Lung biopsy	2 (5.9%)	
	Feeding tube insertion	2 (5.9%)	
	Abdominal abscess drain insertion	3 (8.8%)	
	Drain review studies	2 (5.9%)	
	Percutaneous cholecystostomy/biliary drain insertion	1 (2.9%)	1 (3.6%)
	Biliary stent insertion		1 (3.6%)
	Percutaneous nephrostomy insertion/change	2 (5.9%)	

A table describing all IR procedures performed at NCID on COVID-19 positive or suspected patients, and IR procedures performed during the SARS-CoV outbreak. Insertion of peripherally inserted central catheters and para/thoracocentesis remain common procedures performed during these infectious diseases outbreaks. An increasing number of General intervention treatments and interventional oncology treatments were performed during the COVID-19 pandemic compared to the SARS-CoV outbreak

## Discussion

This study highlights key trends with the potential to evoke change in current proposed procedural guidelines [17–19]. The demand for IR procedures in COVID-free patients remains high during this pandemic. While postponement of procedures might be feasible in a relatively shorted outbreak such as SARS-CoV, these measures might prove detrimental to patients as the pandemic is slated to persist. The adherence to standard infection control protocols and strategic deployment of resources (elaborated in the subsequent paragraph) proved effective in maintaining operational capability and controlling disease spread. The vast majority of patients undergoing IR procedures were in fact COVID-free. This emphasizes the need for procedural planning to consider the needs of COVID-free patients. Key differences exist between the SARS-CoV outbreak and the COVID-19 pandemic. There were notably less infected patients during the SARS-CoV outbreak (*n* = 238) [23, 24]; compared to the COVID-19 pandemic (*n* = 47,126). The SARS-CoV outbreak lasted approximately three months in Singapore, compared to the current pandemic into its 6th month. Unlike the COVID-19 pandemic, our institution treated SARS-CoV patients solely for the entire outbreak duration. This limited us to comparing IR procedures between the two outbreaks to only the infected patients (Table 3). Comparing the differences in IR procedures between the two outbreaks, we note the lack of a tandem increase in the number of IR procedures performed for the infected patients despite the increase in patient load. We postulate this to be due to the intensivists performing more critical care procedures (e.g., venous accesses and thoracocentesis) in this pandemic compared to the SARS-CoV outbreak. In addition, more complex IR procedures were performed for patients' pre-existing condition (e.g., hepatic tumor embolization) and previously unknown coronavirus related manifestations (e.g., thoracic endografting for aortic thrombosis with distal embolization).

To balance infection risks and timely delivery of care for the COVID-free patients, we utilized the strategy of "list compression". Firstly, Tier 1 cases were deferred for an additional month, and this created vacancies in the schedule. The vacated schedules were then utilized to bring forward Tier 2 cases, effectively reducing their waiting times. This is reflected in similar total IR caseloads between the two study periods (Table 1). This strategy is consistent with treatment prioritization principles [17, 19], optimizes available resources, and averts post-pandemic rebound of case backlogs. Additionally, it allowed expedited treatment of Tier 2 cases under the specter of the sudden cessation of services, should the pandemic worsen. A caveat to this approach given the fluid nature in a pandemic, was that majority of our Tier 2 cases were outpatient-based treatments that do not burden inpatient resources and could be rescheduled at short notice. The only exception to our approach was to indefinitely defer treatments (e.g., aortic arch repairs) deemed to have a high potential of occupying intensive care resources, until a suitable time after the pandemic's peak. Furthermore, manpower diversion to COVID-19 related efforts was mitigated by additional staffing, due to curtailment of research and teaching activities, as well as overseas travel/vacation restrictions. Non-essential travel of physicians and patients was mitigated by telemedicine, including the use of telemedicine for nuclear medicine physicians for the administration of radioembolization [25].

Since the pandemic began, calculated measures were made to manpower deployment to allow the Interventional Radiology team to function at full pre-pandemic capacity. The 12 Intervention Radiologists were divided into 2 teams, with only 1 team performing procedures at any week. Manpower for nurses and radiographers in the Interventional Radiology team were increased during the pandemic (12 nurses and 14 radiographers during the pandemic versus 10 nurses and 13 radiographers pre-pandemic). These steps were established to cope with additional processes in infection control. The manpower deployment is summarized in Table 4.

**Table 4:** Manpower deployment during the COVID-19 pandemic

	Manpower count (duration of deployment in weeks)			
	NCID IR Suite	Non-NCID IR Suite	Non-IR out-rotation	Total number
Doctors	1 (1)	6 (1)	5 (1)	12
Radiographers	4 (1)	5 (2)	5 (1)	14
Nurses	4 (1)	6 (1)	2 (1)	12

Stoppage of leave, academic and teaching activities resulted in increased manpower available for deployment. No intermix between the various manpower groups was permitted

A table summarizing the manpower deployment and rotation schedule of Intervention Radiologists, Radiographers and Nurses in the Intervention Radiology team in the NCID IR suite, non-NCID IR suite and deployment to the diagnostic imaging sectors of the Radiology Department. Stoppage of leave, academic and teaching activities resulted in increased manpower available for deployment. No intermix between the various manpower groups was permitted

Limitations exist to the broad application of our results. Unlike many other countries, our mortality and need for intensive care among COVID-19 patients remain the lowest in the world [4]. While we modeled our capacity preparation on countries that experienced COVID-19 peaks before us (such as Italy and the United Kingdom), our healthcare system was never overwhelmed. Additionally, the NCID with its IR facilities, served as a surge capacity and a physical buffer for the first 15 weeks into the pandemic, a resource not available in most institutions. Our practice, particularly the infection control policies and related resources, were also shaped by our prior experience as the designated hospital for the SARS-CoV outbreak in 2003 and the H1N1 pandemic in 2009. Our experience, therefore, can be expected to be different from other centers. Potential bias in the patient load exists, particularly in the postponement of procedures by patients in view of the pandemic. These phenomena could affect the various sub-group analyses performed.

To conclude, our study showed that COVID-19 patients accounted for a small fraction of IR procedures, while the demand for IR procedures for the COVID-free patients remained high and similar to pre-pandemic levels. Application of treatment prioritization guidelines, when combined with standard infection control measures, can allow safe and timely delivery of care to COVID-free patients in caseload volumes similar to pre-pandemic levels. These findings should be considered in the operational planning of a sustainable care delivery model in IR, considering the expected prolonged COVID-19 pandemic and in subsequent infectious disease outbreaks.

## Supplementary information

**Additional file 1:** A table detailing all 3 tiers of the IR-PAS classification.

## Data Availability

The datasets used and analyzed during the current study are available from the corresponding author on reasonable request.

## Abbreviations

COVID-19: Novel coronavirus
IR-PAS: Interventional Radiology—Procedure Acuity Scale
NCID: National Centre for Infectious Diseases
SARS-CoV: Severe Acute Respiratory Syndrome
