# Editorial Expression of Concern: Trends of interventional radiology procedures during the COVID-19 pandemic: the first 27 weeks in the eye of the storm

**DOI:** 10.1186/s13244-025-02141-z

**Published:** 2025-11-19

**Authors:** Guo Yuan How, Uei Pua

**Affiliations:** Tan Tock Seng Hospital, Singapore, Singapore

**Editorial Expression of Concern to: Insights into Imaging (2020) 11:131**

10.1186/s13244-020-00938-8; published online: 09 December 2020

The Editor-in-Chief is issuing this editorial Expression of Concern to alert readers that the protocol approved by the ethics committee for this study was based on an estimated 3000 patients. However, the automated search function used by the authors actually included the records of 7506 patients in the analysis. The ethics committee has granted retrospective approval for 7506 patient records. The authors agree with this statement.

