# Supplementary material for: Trends of interventional radiology procedures during the COVID-19 pandemic: the first 27 weeks in the eye of the storm
Source: Insights Imaging. 2020 Dec 9;11:131. doi: 10.1186/s13244-020-00938-8 (PMC7724451; doi:10.1186/s13244-020-00938-8)
Supplement: Supplementary file 1 — Additional file 1: A table detailing all 3 tiers of the IR-PAS classification. [file 13244_2020_938_MOESM1_ESM.docx]

**ELECTRONIC SUPPLEMENTARY MATERIAL**

Interventional Radiology - Procedure Acuity Scale (IR-PAS)

| Tiers/Description | Definition | Examples | Action |
| --- | --- | --- | --- |
| Tier 1 | Low acuity procedure  Outpatient procedure Not life-threatening illness | Non-Vascular:   - Thyroid Biopsy (incidental finding) - Botox injections - Cosmetic IR - Fallopian tube recanalization - Select renal ablations (e.g. T1a)* - Routine tube/drain change - Image guided liver biopsy for abnormal LFTs - Percutaneous access for nephrolithotomy (in conjunction with Urology) - LP for non-infection/neurologic causes (not including intra-thecal chemotherapy) - Hysterosalpingogram - Myelography - Interventional Pain procedures like trigger point injections, joint and facet injections    Vascular:   - Incidental Vascular anomaly/malformation - Asymptomatic or mildly symptomatic May-Thurner syndrome - IVC filter removal - Varicose veins, GSV ablations - EVAR - AAA < 6.5 cm - Peripheral Angiogram and intervention for claudication - Lower extremity venous interventions for symptomatic spider veins or superficial vein incompetence - IVC Filter removal - Uterine fibroid embolization - Pelvic congestion embolization - Prostate artery embolization - Venous sampling - TIPS for Ascites - Central venous catheter/port removals for completion of treatment    *Consider shared decision-making via virtual multi-disciplinary tumor board discussion | Postpone procedure |
| Tier 2 | Intermediate acuity procedure  Not life threatening but potential for future morbidity and mortality. Requires in hospital stay | Non-Vascular:   - Joint aspiration for infection - Percutaneous fluid collection drainage for infection without septic shock – consider antibiotic trial first - Cancer Diagnostic procedures (biopsies, aspiration) * - Cancer therapy (locoregional therapy) * - Tube change for malfunction or leakage - Gastrostomy/GJ tube placement for nutrition - Bone Augmentation/ Vertebroplasty/Kyphoplasty/ Sacroplasty - Thoracentesis/Chest tube for dyspnea - Therapeutic Paracentesis     Vascular:   - Venous Thoracic outlet syndrome intervention for mild symptoms - Endovascular management of asymptomatic peripheral and non-aortic intrabdominal aneurysm - AAA > 6.5cm - TAA > 6.5cm - Fistula/Dialysis access interventions for suboptimal function - Chronic mesenteric ischemia interventions - Peripheral Angiogram and intervention for chronic limb threatening ischemia – rest pain or tissue loss - Massive iliofemoral DVT without phlegmasia - Venous intervention for ulcers in lower extremity - IVC filter placement in low risk patients - Central Venous Catheter or PICC line placement     *Consider shared decision-making via virtual multi-disciplinary tumor board discussion | Postpone procedure if possible |
| Tier 3 | High acuity procedure | Non-Vascular:   - Percutaneous fluid collection drainage for septic shock - Percutaneous Nephrostomy for septic shock - Percutaneous Cholecystostomy/Biliary drain for septic shock - Transplant rejection solid organ biopsy - Lumbar puncture for infection, acute neurologic change or intrathecal chemotherapy     Vascular:   - Trauma interventions - Pulmonary Embolism Therapy - Stroke interventions - Acute hemorrhage interventions/embolization - Arterial lysis/thrombectomy for acute ischemia - EVAR/TEVAR for ruptured or symptomatic TAAA/AAA - EVAR/TEVAR for dissection with rupture or malperfusion - Symptomatic Carotid artery stenosis intervention - Symptomatic acute mesenteric ischemia intervention - PAD for Limb ischemia: progressive tissue loss, acute limb ischemia Intravascular management of PAD leading to acute limb ischemia - Endovascular management of symptomatic peripheral and non-aortic abdominal aneurysms - Endovascular management of pseudoaneurysm – expanding, iatrogenic - Fistula/Dialysis access interventions for thrombosis | Do not postpone |
